# Readiness to Accept Genetic Testing for Personalized Medicine: Survey Findings on the Role of Socio-Demographic Characteristics, Health Vulnerabilities, Perceived Genetic Risk and Personality Factors

**DOI:** 10.3390/jpm12111836

**Published:** 2022-11-03

**Authors:** Anja Leppin, Jesper Bo Nielsen

**Affiliations:** 1 Unit for Health Promotion Research, Department of Public Health, Faculty of Health Sciences, University of Southern Denmark, 6705 Esbjerg, Denmark; 2 Unit for General Practice, Department of Public Health, Faculty of Health Sciences, University of Southern Denmark, 5000 Odense, Denmark

**Keywords:** personalized medicine, precision medicine, pharmacogenetic testing, public acceptance, socio-demographic characteristics, health-related behaviors, subjective health, perceived genetic risk, personality factors

## Abstract

Studies from various countries have shown that majorities would accept genetic testing for personalization of treatment, but little is known about differences among population subgroups. The present study investigated whether readiness to accept a hypothetical cost-free offer of genetic testing to personalize treatment depends on socio-demographic characteristics, health-related vulnerabilities, personal dispositions, and prior awareness about personalized medicine. The study was based on a cross-sectional survey design. Out of a representative initial sample of 50–80-year-old Danish citizens (*n* = 15,072), *n* = 6807 returned a fully answered web-based questionnaire. Socio-demographic data were added from a national registry. Data were analyzed by multivariable logistic regression. A large majority of respondents (78.3%) expressed their readiness to be tested. Rates were higher in men, younger persons, and those with higher income. Additionally, ex-smokers and obese persons as well as those less satisfied with their health and respondents who perceived a personal genetic risk were more interested, as were those with higher internal health control, higher extraversion, higher emotional stability, and those who had not heard about this option before. Further research should investigate the specific concerns among population subgroups which need being addressed by systematic communication efforts in a clinical but also a broader public health context.

## 1. Introduction

Genetic testing—as a cornerstone of personalized or precision medicine—has found entrance into clinical guidelines and is gaining ground in clinical practice as part of a larger patient-centered approach [1,2,3,4]. The terms ‘personalized medicine’ or ‘precision medicine’ have been defined in different ways, and no formal consensus about a definition has been reached yet [5,6]. However, common assumptions are that health outcomes can be improved by “stratification and timing of healthcare” [5], and that this stratification process for specific population subgroups will be enabled by “biological information and biomarkers on the level of molecular disease pathways, genetics, proteomics as well as metabolomics” [5]. Areas of application range from assessing disease and therapy risks to “screening, prognosis, diagnosis, treatment selection, and surveillance or monitoring” [6].

To the extent that interindividual genetic variation leads to differential responses to drug treatments, genetic/genomic assessment may provide guidance for drug choice and dosage and thereby make treatment more efficient [4,7]. Typical areas of application are cancer treatments (for instance breast, lung, colon), secondary prevention of stroke and coronary heart disease, such as in anticoagulant treatment with Warfarin or Clopidogrel, treatment for psychiatric conditions, but also polypharmacy-related side effects [4,7,8,9,10]. 

Yet, many open questions remain, from cost effectiveness to clinical utility and public health implications [6,11,12,13,14]. Additionally, optimal delivery models for these still comparatively new approaches are debated. Implementation challenges have been identified on different levels and include economic, legal, ethical as well as operational aspects [15]. In particular, there is a need to develop effective modes of information delivery for different target groups as well as education/training programs for the health care professionals whose task it is to inform their patients and initiate a shared-decision-making process [15,16,17]. To support this communication process, it is essential to ascertain what populations already know about personalized medicine, how they view this option, and to which extent they are prepared to accept it [15,18,19].

Findings from focus group and interview studies, conducted in different countries and contexts with general populations as well as patient groups, suggest that expectations about the benefits of personalized medicine tend to be high. Many believe in an improvement of health care and treatment efficacy as well as occurrence of fewer/milder side effects [20,21,22,23,24]. At the same time, widespread concerns have been identified, the most important of which are related to privacy and data protection, coverage of costs for testing and targeted treatment. Further, there seem to be prevalent concerns about a possible overreliance on genetic results by physicians and worry that stratification could lead to inequalities and to rationing of treatment access [20,21,23,24,25,26,27,28].

Evidence from large-scale quantitative surveys conducted in Canada, Denmark, Germany, Japan, Korea, Singapore, and the US are largely in line with reports from these focus group studies. Most surveys investigated pharmacogenetic testing and found that majorities hold positive attitudes and/or would personally accept a genetic test aimed at targeting medication [29,30,31,32,33,34,35,36,37,38,39,40]. However, little is known yet about the homogeneity of such findings, that is to which extent different population segments agree or differ in their views of personalized medicine. Some studies have investigated the role of socio-demographic factors, but findings are inconsistent [29,30,31,33,34,35,36,37,40]. 

More specific characteristics, such as a person’s health status or health risk factors, or a person’s sense of control over their own health have found even less attention in research on acceptance of personalized medicine. Knowledge about such differences might be important though because it may provide valuable input for an improved communication process about benefits and limitations of personalized medicine with the public as well as patients.

One factor which can be expected to influence acceptance is health status. Current experience of health problems is likely to make a need for effective treatment more personally salient and lead to a stronger focus on potential benefits than on problems of novel treatments. In a similar vein, those aware of personal behavioral or genetic risk factors could also have a stronger motivation to accept new strategies to optimize treatment, since subjective experience of risk is likely to increase motivation to search for and adopt protective measures [41,42]. Those who experience no health problems and/or perceive themselves at low risk, on the other hand, might view the issue from a larger psychological distance. Their attitudes towards personalized medicine might therefore reflect general worldviews about genetic technologies rather than personal hopes or concerns and thus show stronger variation.

Additionally, motivation to adopt new medical technologies may be driven by personal-psychological dispositions. For instance, those with a strong sense of personal control over their health could be expected to have a stronger motivation for optimizing their treatment. Among more general personality traits, higher habitual ‘openness’ may lead to a stronger interest in trying new scientific/medical-technological options. At the same time, individuals who are more outgoing, energetic, and approach/action-oriented (‘high extraversion’), and those with high levels of ‘emotional stability’ may habitually tend to perceive benefits from novel treatments rather than worry about possible negative consequences or side effects and therefore be more accepting [43].

Yet, another factor which can be expected to play a relevant role in this context is prior awareness or knowledge about personalized medicine. Some studies have already investigated such an influence, but findings are again discrepant [31,38,39,44]. On one hand, familiarity with personalized medicine may predispose people towards feeling more comfortable with these technologies and thus make them more ready for adoption. On the other hand, knowledge may not only result in better understanding of benefits but also of limitations and unresolved issues in an area with many uncertainties and with incomplete and/or insufficiently communicated regulation. In this case, the expected consequence would be a higher level of caution or reluctance to accept such procedures.

In the present study, we investigated the role of such personal characteristics for readiness to accept a hypothetical cost-free offer of a genetic test aimed at targeting and optimizing treatment. Study participants were 50–80-year-old Danish citizens. This age group is relevant in the given context, since it is particularly likely to need medical treatment that might also involve decisions about genetic targeting.

Specifically, we tested whether testing readiness was independently associated with the following factors: general socio-demographic background (sex, age, education, income), health risks/vulnerabilities (smoking, alcohol intake over recommended levels, insufficient physical activity, obesity, perceived genetic risk), current subjective health status (satisfaction with health, daily medication intake), health-related and more general psychological dispositions (internal health locus of control, disposition to take health risks and the personality factors ‘emotional stability’, ‘extraversion’ and ‘openness’), as well as prior awareness/knowledge about the option of personalized medicine.

## 2. Materials and Methods

### 2.1. Sample and Procedure

The study was based on a cross-sectional survey design, using a representative sample of 50–80-year-old Danish citizens collected by Statistics Denmark. (Statistics Denmark is a Danish governmental agency under the Ministry of the Interior and Housing with the central responsibility for creating statistics on the Danish society.)

**Figure 1:**
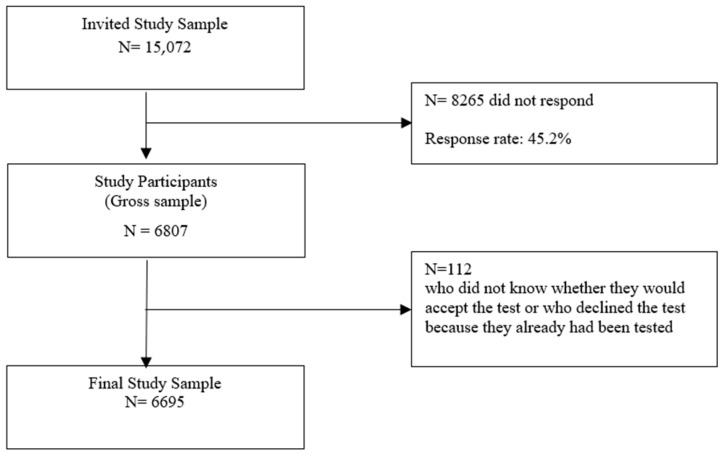
Flow chart on Study Sample Size.

Initial sample size was *n* = 15,072. Contact was established via the national Danish “digital mailbox system” used for official communication between public agencies and citizens. Those who were able to read and understand Danish as well as agreed to participate were provided with access to a web-based, standardized questionnaire. These self-reported data were combined with socio-demographic information from the national Danish citizen’s registry. Two reminders were sent through digital mail. A total of *n* = 6807 persons (45%) returned a completed questionnaire (gross sample) (see Figure 1).

### 2.2. Measurement

#### 2.2.1. Outcome Variable

Readiness to accept a hypothetical free-of-charge offer of a genetic test aimed at targeting treatment was assessed as follows: First, an introductory question—presented under the headline of “personalized medicine”—asked respondents whether they had already heard about use of genetic tests to improve treatment for some diseases. This was followed by a brief description: “A genetic test is easy and pain-free to do by sending a saliva sample to a laboratory. In case of need the results allow your physicians to adapt your treatment for some diseases more easily and more quickly. These diseases can be both serious or less serious. Health gains are rarely huge but typically treatment might work more quickly for you and there will be fewer side effects”. This was followed by the question: “*Would you accept a free-of-charge offer of such a test*?” Response options were “yes”, “no” and “don’t know”.

#### 2.2.2. Exposure Variables

Standard socio-demographic information about sex, age group (50–60, 61–70, 71–80), highest level of educational attainment and income came from the national Danish registry. Educational level was trichotomized into “less than 11 years”, “11–13 years”, and “over 13 years” of school education. Income was assessed in terms of the OECD scale for “equivalized personal disposable income”, which uses a weighting factor for number of persons in household to determine personal income after taxes. The initial five income categories were collapsed into three groups: "below 33.333 EUR", "33.333–46.666 EUR" and "over 46.666 EUR".

All other exposures were assessed by self-report questions. Satisfaction with general health status was measured by a single item: “*How satisfied are you with your current state of health?*” which was to be rated on a 10-point scale from “not satisfied at all” to “highly satisfied”. Responses were categorized into: “low satisfaction” (0–3), “medium satisfaction” (4–6), “high satisfaction” (7–10).

Smoking status was assessed by the question: *“Do you smoke?”,* which had three response options: “current smoker”, “quit smoking”, and “never smoked”. Another single item tapped into alcohol consumption: *“How many units of alcohol do you usually drink in a week?*” Answers were categorized into: “none”, “1–14/1–7 units per week” and “over 14/7 units per week” for men and women, respectively, following recommendations by the Danish National Board of Health [45].

Physical activity was measured by one question: *“In a typical week, on how many days do you engage in exercise for at least 30 min at moderate to high intensity?”* Further explanation provided was: “Moderate physical intensity is physical activity where you are slightly out of breath but can still talk to others”. Responses ranged from 0–7 days. We used a cut-off of ≥ 5 days/week as criterion based on the guidelines for adults by the American College of Sports Medicine and the American Heart Association [46].

Self-reported weight (kg) and height (meters) were used to calculate Body Mass Index. Scores were categorized into three levels: “<25” (underweight/normal weight), “25–29.99” (overweight), and “≥30” (obese) according to WHO criteria [47].

Health locus of control was assessed by the six item-Internal Health Locus of Control-Scale (MHLC-Internal Form A) tapping into a personal sense of control over health outcomes [48]. An example item is “*The main thing that affects my health is what I myself do”.* All items were presented with a 6-point Likert scale from “completely disagree” to “completely agree”. Item scores were summed up and divided by six. If only one value per respondent was missing, it was replaced by the mean of the remaining items for the respective person. Cronbach’s alpha for the scale was 0.84. The distribution was dichotomized at the median (MD = 3.67) into those “low to medium” and “medium to high” in internal control over health.

A general tendency for health-related risk taking was measured by the question *“How do you evaluate your willingness to take a risk related to your health?”* The response scale ranged from 0 to 10. Answers were categorized into three groups: “no willingness to take health risks” (0–1), “low to medium willingness to take health risks” (2–5) and “high willingness to take health risks” (6–10).

Furthermore, respondents were asked about perceived personal genetic vulnerability by one question, *“Do you think that you have a genetic vulnerability, which means you have a greater risk for some diseases than other people?”* Answer options were “yes”, “no” and “don’t know”.

Another single item assessed prior awareness about the possibility to use genetic tests for treatment stratification: “It is possible today to conduct a genetic test to improve treatment for a number of diseases. Have you heard about such genetic tests”? Answer options were “yes”, “no” and “don’t know”.

Personality dispositions were measured with the Ten-Item Personality Inventory (TIPI) [49], a widely used, brief measure of personality. Each dimension is assessed by two items and each item is scored on a 7-point Likert scale ranging from 1 = disagree strongly to 7 = agree strongly. Sum scores for the dimensions were divided by 2. This resulted in scores from 1–7 where higher scores indicated higher levels of the respective personality trait.

### 2.3. Statistical Analyses

Analyses were conducted with IBM SPSS version 26.0 [50]. All associations were first tested on a bivariate level via Chi-Square tests. Subsequently all variables were jointly entered into a multivariable logistic regression equation to test which of the exposure variables were independently associated with readiness to use a genetic test for treatment stratification. For individual predictors, *p*-values < 0.01 were considered significant.

## 3. Results

### 3.1. Sample Characteristics

The initial gross sample included *n* = 6807 participants. Slightly more than half of the respondents were female. Almost equal proportions of approximately 39% and 37%, respectively, were between 50–59 and 60–69 years old, while the group of 70–79-year-olds made up the remaining quarter (see Table 1). Around 37% had a college/university education, and approximately one third each a low, medium, and high personal income (see Table 1). While the distribution of some characteristics matched those of the national population aged 50–80 (sex, distribution of residences across the country), deviations were noted for others. Citizens 50–60 years old were under- and those 61–70 overrepresented by about 4% each. The largest differences occurred for level of education. There was an underrepresentation of about 8% of those with the lowest and a matching overrepresentation of those with the highest education (see Table 1). Additionally, the present sample had about 7% less respondents from the lowest income bracket, while those from the highest income group were overrepresented by 5% compared to the reference population. Finally, citizens with a birthplace outside of Denmark were underrepresented compared to the reference population.

### 3.2. Awareness of Personalized Medicine

62.4% had already heard about genetic testing for treatment stratification, while 37.4% had not. Very few (0.2%) did not know or could not remember whether they had heard about it. A multivariable logistic regression, excluding the small group of those who had said they did not know whether they had heard about such a test (*n* = 16), showed that awareness rates were higher among women than men (68.9% vs. 55.2%; OR = 1.84; CI = 1.66–2.03). Additionally, compared to 71–80-year-olds (58.2%), larger proportions were aware about this option among the 50–60-year-olds (62.6%) and those 61–70 (65.4%), but only the latter difference was significant (OR = 1.20; CI = 1.05–1.38). Further, while 51.9% among the lowest educational group had already heard about genetic testing for personalized medicine, these rates were higher in the groups with longer education (11–13 years: 57.6%; OR = 1.26; CI = 1.10–1.44; over 13 years: 73.6%; OR = 2.35; CI = 2.03–2.72). A similar gradient was found between income groups. While 56.1% from the lowest income group reported being aware of the testing option, the rates were 63.6% in the medium income group (OR = 1.28; CI = 1.13–1.45) and 69.2% in those from the highest income bracket (OR = 1.49; CI = 1.30–1.70).

### 3.3. Readiness for Treatment-Related Genetic Testing

A large majority of respondents (78.3%) expressed readiness to make use of free-of-charge genetic testing, if it were offered to them to improve treatment. About one fifth (20.3%) declined and 1.4% (*n* = 93) responded that they were not sure/did not know what they would do. Respondents who declined the offer were further asked to indicate their reasons by choosing one or more options from a pre-set list. The response selected most often (72.9%) was not wanting to find out that one might develop a specific disease later in life. Almost a quarter (23.6%) said “no” because they did not expect any personal benefits from being tested. Uncertainty about how others might use one’s test results was an issue for 10.6%, while 4.6% said tests such as these could not be trusted. Only 1% indicated that they already had participated in such a test. The option “other reasons” was selected by 8.1%.

To explore differences between socio-demographic subgroups logistic regressions, with all other socio-demographic variables adjusted for, were conducted for reasons chosen by at least 10% of decliners. Women were less likely than men to claim lack of personal benefits (OR = 0.52; CI = 0.40–0.67) as a reason for their disinterest or to indicate uncertainty about how others might use their data (OR = 0.62; CI = 0.43–0.88), but more often did not want to learn about potential future diseases (OR = 1.69; CI = 1.32–2.17). In relation to age, potential misuse of data was more often named as a reason for non-acceptance by 50–60-year-olds as compared to those 71–80 years (OR = 3.0; CI = 1.82–4.86). Younger groups, but particularly the 61–70-year-olds, were more likely to name not wanting ancillary information about disease risk than the oldest, aged 71–80 years (OR = 1.46; CI = 1.08–1.98).

Likewise, those with the highest-level education more often named unwanted information about future diseases as a reason for rejecting the offer than those with the shortest education (OR = 1.55; CI = 1.12–2.14). Additionally, they were more likely to claim potential misuse of data as a cause of their disinterest (OR = 1.80; CI = 1.03-3.14). Further differences were noted between income groups. Those with medium and higher income were less likely than those with a low income to point out lack of personal benefits as a reason (OR = 0.71; CI = 0.52–0.98/OR = 0.63; CI = 0.45–0.88) but more likely to indicate they did not want to learn about future diseases (OR = 1.52; CI = 1.12–2.06/OR = 2.23; CI = 1.59–3.11).

Subsequent analyses excluded respondents who (a) had said they did not know whether they would accept the genetic test (*n* = 93) or (b) indicated that they would not accept the test and further stated that this was because they had already taken such a test or did not know/were not sure whether they had already done so (*n* = 19). This resulted in a final sample of *n* = 6695 which deviated only minimally (at most 0.3%) from the gross sample in terms of age, sex and education (see Table 1).

Bivariate associations between readiness to be tested and sample characteristics are shown in Table 2. Actual sample sizes differed between *n* = 6695 and *n* = 6572 for individual analyses due to varying numbers of missing values/’don’t know-responses’. For some variables there were no missing values (all socio-demographic characteristics, awareness about and readiness to receive a genetic test, perceived genetic vulnerability, smoking and alcohol consumption). For physical activity, satisfaction with health, and health risk taking tendencies the number varied between 2 and 12. For the three personality dispositions 12–20 missing values were noted, while 47 missing values were recorded for the 6-Item Internal Health Locus of Control Scale. The highest number of missing values occurred for Body Mass Index, where *n* = 125 respondents did not fill in the necessary information to compute the variable.

Table 3 presents crude as well as adjusted Odds ratios from a multivariable logistic regression model. All variables were entered simultaneously into the prediction equation for readiness to take up genetic testing in the context of medical treatment (no/yes).

Sex, age, and income were independently related to acceptance of a hypothetical free testing offer. Females had about 30% lesser odds for acceptance than males. Further, willingness to be tested seemed to decrease with age. However, only the difference between the youngest (50–60) and the oldest group (71–80) was significant. Finally, those with medium and higher income were significantly more likely to respond positively to the testing offer than those from the lowest income bracket.

Alcohol intake and physical activity were unrelated to testing readiness. However, ex-smokers were more willing to accept the test than those who were currently smoking. Additionally, obese respondents (BMI = 30 or above) were more inclined to say ‘yes’ to the test compared to those being normal/underweight.

Taking medication daily did not make a difference. However, those with medium or high satisfaction regarding their health had about 25–35% lesser odds to express an interest in testing compared to those who were least satisfied. Another highly significant difference was noted for perceived genetic risk. Compared to those who did not believe they were genetically vulnerable, those who did not know whether they had a genetic disease disposition or those who were convinced they had such a vulnerability had higher odds for test acceptance (30% and 90%, respectively) (see Table 3).

A habitual tendency to take health risks did not make a significant difference. However, having a higher as compared to a lower health locus of control increased the odds of accepting the hypothetical offer by 20%. Higher extraversion and emotional stability, but not higher openness, were significantly positively associated with higher willingness to take the test.

Finally, those who said they had heard about the testing option to improve medical treatment had about 40% lesser odds to react positively compared to those who said they had not heard about this option before (see Table 3).

## 4. Discussion

Slightly over 60% of Danish citizens aged 50–80 were aware of the option of personalized medicine - a rate which seems to have nearly tripled compared to a survey conducted in 2004 [32]. A direct comparison is difficult though since the 2004 survey included all age groups among the adult Danish population instead of only the older segment.

Awareness levels differed among population subgroups. Thus, higher percentages among women than men had heard about personalized medicine, which is consistent with findings from other countries, such as the US [31] as well as earlier Danish findings from 2004 [32]. In general, women have been reported to have a stronger interest in health topics and to engage more often and more intensely in health information-seeking behavior than men [51,52,53].

Further, higher education and higher income were associated with awareness about personalized medicine, which similarly is consistent with survey results from the US [31]. Further, Danish survey findings from 2016 also showed positive associations of education with self-evaluated knowledge about pharmacogenomic testing [37]. Citizens from higher socio-economic strata may generally have stronger health-related interest/motivation, higher health literacy and engage more in health-related information-seeking, which then also covers more specific areas of health and health care.

Nearly 80% responded positively towards the hypothetical offer of a genetic test for treatment targeting. Majorities in favor of pharmacogenetic testing have also been reported from smaller- or larger-scale population surveys among varying age groups in other countries [31,40,54]. More specifically, the findings are in line with those of the national Danish survey from 2004, which reported that 79.1% thought that "society needs pharmacogenetics” [32], but also with results from a 2016 Danish survey among 1005 citizens 18+, which found that 83% would accept a genetic test in the context of medical treatment [37].

However, beyond this generally favorable view, some degree of variation among population subgroups became visible. Thus, acceptance was lower in the oldest (71–80 years) compared to the youngest group of 50–60-year-olds. Less motivation for pharmacogenetic testing or lesser positive attitudes in older age groups have also been reported from focus group studies [20,55] and from a population survey conducted in Japan [30], while surveys from the US and Singapore reported no age differences [31,40]. Direct comparisons with the present study are difficult, however, since most other studies involved a broader age range of adults, while the present study focused only on the older population segment. However, Chapdelaine et al., in a Canadian survey on elderly primary care patients, also found lower willingness to be tested for treatment targeting among the older segments [29]. Older persons might have more cautious worldviews regarding new technologies in general [56], but also may have longer experience with certain treatments and might experience reluctance to change familiar procedures comparatively late in life.

Interest in the hypothetical test offer was also lower among women. Some prior studies similarly reported lesser acceptance or more fearful attitudes regarding treatment-related genetic testing in females. This was for instance shown in a German study on asthma and COPD patients by Rogausch et al. [34] or a study with Chinese Singaporean patients in the context of Warfarin-related pharmacogenetic testing [40]. In contrast, other studies found only partial or no sex differences [29,30,31,33]. In the present study, the higher reluctance among women was not due to a lack of perceived benefits or concerns about potential abuse of data, since they endorsed these reasons for declining the offer less often than did men. Instead, women seemed to worry more about unwanted information about diseases they might develop later in life. The identification of this particular concern is consistent with the prior Danish survey study from 2016 where fewer women than men indicated a desire to receive information about genetically based disease risks from pharmacogenetic tests [37].

Further variability was found in relation to economic status. A higher income went along with a more pronounced test interest. This factor has—to our knowledge—not been investigated in larger scale surveys among the general public. However, focus group findings from different countries have suggested that financial costs are among the more often raised concerns regarding personalized medicine [20,23,24,25,26,27,28,55]. It is interesting though that such worries may be triggered even in “welfare states” with universal health coverage, such as Denmark, and even though the test had been described as cost-free. This observation is supported by findings from citizen panels conducted in Denmark in 2016. Who should shoulder the costs of personalized medicine was a highly prevalent concern, and there was a broad-based consensus that these new medical options should not be too expensive for society [37]. Such concerns, which are likely to be stronger in those with lesser income, could be due to an anticipation of future changes in favor of out-of-pocket payments or increased taxes to finance a growing health care budget. In a related vein, other findings from Denmark have shown that lower income was associated with perception of more structural barriers to preventive lifestyle change [57].

Those with behavioral risk factors and/or already existing health problems were expected to react to these vulnerabilities with a stronger inclination towards test acceptance because personal relevance of the hypothetical testing situation might be higher and a felt need to “compensate” potential risks stronger. In line with this, ex-smokers had a significantly higher interest in testing compared to non-smokers. Stopping smoking might reflect a higher awareness of a health risk as well as a generally stronger health protection motivation compared to those who still smoke. Similarly, obese respondents were more interested in the offer than the non-obese. Obese persons may already have encountered problems with drug dosage due to their condition, since (severe) obesity can affect pharmacokinetics and -dynamics [58,59]. Prior experiences with drug dosage problems may thus have sensitized these persons towards their higher need for optimized treatment.

Levels of alcohol consumption and physical activity, on the other hand, did not affect testing acceptance. A possible, if speculative explanation for this difference between the specific behavioral risks could be that the strong focus of public debates in Denmark on smoking and nutrition/obesity in the recent decade has specifically sensitized smokers and obese persons towards being “at risk”. This perception might have led to a stronger subjective need for especially effective treatment. Alcohol consumption and comparatively low-level physical activity on the other hand might still be seen as more normative and accordingly less “risky” among the older population segments. Another and different type of consideration is that the assessment of alcohol consumption as well as physical activity level by a one-item self-report frequency measure may have led to a higher level of misclassification than the comparatively easier to identify characteristics ‘smoking status’ and ‘weight/height’.

Perceived genetic risk and/or family history of a disease have been found to increase preparedness for predictive testing [60,61,62]. In line with this, we also identified a positive association of perceived personal genetic risk with interest in personalized medicine. Those who were unsure whether they were at increased risk due to genetic factors, and even more so those who were certain they had such a vulnerability, were more likely to be interested in the test. Perceived personal risk is generally assumed to lead to a stronger motivation towards seeking health protection [41,42]. Moreover, a sense of carrying a genetic risk may also involve a stronger subjective credibility of the concept of personalized medicine. People who are aware of a family history of certain diseases may also be more likely to believe in the value of assessing genetic sensitivity to specific treatments. The cognitive schemata of genetic risks they already possess can make any new information about the relevance of genetic factors, i.e., treatment, more accessible, believable, and easier to process [63]. Further, based on a “representativeness heuristic” [64], the flip side of conceiving oneself as a (prototypical) ‘genetically susceptible’ or ‘vulnerable’ person, may be the expectation that one might also profit more from genetically targeted treatments.

Consistent with a ‘vulnerability hypothesis’, we also found that those who already experienced health problems, indicated by low and medium satisfaction with current health status, were more inclined to react favorably to the testing offer. In a similar vein, Chan et al. (2014) reported that patients’ willingness to undergo genetic testing for drug targeting increased with the number of chronic diseases [40]. Own current negative experience might increase subjective relevance of effective treatment and create heightened motivation to accept a novel approach, such as personalized medicine.

However, the picture may be more complex, as suggested by a US study. Citizens who rated themselves as being in excellent or good health were less accepting of pharmacogenetic testing only when the targeted side effects were presented as “mild”. When side effects were presented as “severe”, this differential effect disappeared, indicating that personal conditions are likely to interact with situational stakes and/or testing functions [31]. Highly compelling situational demands might thus wipe out or reduce differential initial preferences in those with better or worse health status.

In contrast to the general health rating, daily medication intake was irrelevant in our study. However, this may have resulted from a too unspecific assessment. While respondents were explicitly asked to not include vitamins or other dietary supplements, inclusion of medications for acute minor ailments with no relevant side effects might have diluted potential effects. Additionally, rather than ask for medication intake per se it may be more useful to ask for experience of adverse drug reactions [31,33,40].

Beside the outlined associations with health risks and health status, it was dispositional factors which made a difference for test acceptance. One such factor is “health locus of control” which so far has mainly been investigated in the context of predictive testing, where a review by Sweeny et al. reported mixed evidence [62]. In the present study, we found that a higher sense of internal control raised readiness to accept the testing offer. A personal sense of control over health/illness is assumed to have a positive influence on health-protective actions. Persons high in control are more likely to actively work for keeping or restoring their health by different kind of measures [48], and this seems to include taking up novel treatment offers. However, we only assessed the internal control dimension without adjusting for “control by powerful others/medical experts”, a subdimension which tends to play a significant role when it comes to medical treatment. Blouin-Bougie et al. (2017) used both dimensions in their study on acceptance of predictive genetic testing and found a significant effect for “powerful others” but not for “internal control” [65].

Significant associations were also found for two of the three investigated personality dispositions, that is emotional stability and extraversion. Both were positively associated with higher readiness for testing, while openness lost significance in the multivariable model. To our knowledge, personality differences have not yet been investigated in relation to acceptance of personalized medicine. Regarding predictive testing, however, studies have reported significant associations between interest in genetic testing for some cancer types and high dispositional optimism as well as low depression [62,66,67]. Both these factors are closely linked to emotional stability. This suggests that persons less inclined to worry and more likely to expect positive outcomes may be more willing to trade off the “risk” of being tested for a chance to reap personal benefits. Similarly, people who are more extraverted, that is generally more outgoing, action-oriented, and spontaneous might spend less time contemplating potential drawbacks and be less concerned about sharing personal genetic information to optimize their own treatment. However, such mechanisms are speculative at this point and await further empirical testing.

Finally, the proportion of those who said they would not accept the offer was higher among those who had already been aware about personalized medicine before participating in the survey, compared to those who had not. This might seem surprising, since familiarity and understanding can be expected to increase interest and acceptance. Thus, Zhang et al. found that comfort levels with pharmacogenetic testing were higher in those with more knowledge [44].

However, several processes might explain the reverse finding in the present study. First, from the perspective of information processing, those with prior information also had had more time to consider not only benefits but also potential downsides. Those, on the other hand, who were newly introduced to the topic may have focused more on the information about potential benefits and thus have felt more inclined to spontaneously accept the offer. Direct reactivity to the informational focus of pharmacogenetic testing was also shown in the public US survey by Haga et al., where 65% were interested in pharmacogenetic testing after having been informed about potential risks, while the rate was 82% after learning about benefits [31]. Second, having heard about personalized medicine is not equivalent with actual knowledge and understanding about potential benefits and limitations. It seems that (partly) incorrect understanding and false expectations about genetics in general and personalized medicine in particular are no uncommon phenomena [37,38,39,68,69]. In the 2016 Danish survey among adults of all age groups respondents were requested to self-rate their knowledge about personalized medicine. Only 8% stated they knew ‘a lot’ or ‘a real lot’, while 33% indicated they knew nothing or only very little [37]. Additionally, in the US survey by Haga et al. (2012), only slightly more than half of those who had said they had heard about pharmacogenetic testing thought they understood its healthcare application ‘very well’ or at least ‘somewhat well’ [31].

We did not collect information about what exactly participants had heard and to what extent they had been exposed to positive versus critical information. This aspect may be crucial though, as Europeans have been reported to generally have a more critical stance towards genetic testing compared to US citizens [70]. Different societal debates in different countries about novel medical technologies and the risk/benefit frames within which they are presented may lead to more positive or negative feeling tones associated with general awareness. At least parts of the population group represented in our study could have been exposed to a somewhat critical societal debate. Results from Danish citizens’ panels on personalized medicine in 2016 showed that many participants had first been confronted with the topic by a TV program on health testing and screening, including genetic testing, which had been aired in the same year. This program, had raised a series of critical questions [37]. Given that public TV in Denmark still has a considerable reach among the age groups included in our study, those who had heard about personalized medicine before might have been exposed to this more critical viewpoint. In this context it is also important to note that a major reason for rejecting the offer was not wanting to be informed about the potential development of certain diseases later in life. Testing in the context of treatment stratification may indeed also identify unrelated disease risks, but the present findings seem to suggest that some citizens believe that this is a quasi-automatic and inevitable consequence. Haga et al. (2013) investigated the US public perspective on ancillary findings and similarly identified a considerable level of concern about non-anticipated disease information [28]. In the future it might be relevant to more closely investigate the mental models and prior beliefs about personalized medicine among different population groups to better be able to address such concerns and worries [69].

### Strengths and Limitations

The present study was based on a large sample (*n* > 6000) aimed at representing the population in the respective age segment. The response rate of 45% is within the normal range for studies using this type of recruitment. Comparisons with the Danish population in the relevant age segment found no large deviations. Yet, it needs to be noted that there was some underrepresentation of those with short-term education, low personal income as well those not born in Denmark. However, participation is unlikely to have been biased by a differential interest in genetic testing in different population groups. The questions were part of a larger study on health-related issues. When consenting to take part participants were not aware they would be asked about genetic testing/personalized medicine.

A limitation is the cross-sectional character of the study, which precludes any causal interpretations. Additionally, the single-item self-report-based assessment of physical activity, alcohol consumption, BMI as well as tendency to take health risks might have restricted reliability and validity.

Further, there could be doubts about the extent to which hypothetical questions about future behavior are valid predictions of actual future behavior. However, in another study, we found no difference in predictors for participation in the national Danish colon cancer screening program by those who already had participated and those who were not yet eligible due to being too young and who instead reported their anticipated willingness to take part [71]. Yet, screening and treatment decisions might not be entirely comparable. Actual decisions about acceptance of personalized medicine may create different qualities and intensities of hope, fear and felt pressures, so more studies about actual uptake in different clinical settings are needed.

Another caveat in relation to the used scenario is its very generic character. Since it was non-specific regarding disease or type of treatment, it cannot be excluded that a focus on severe or life-threatening diseases would have generated more uniform responses. Similarly, the specific purpose of the testing (drug effectiveness or mild or severe side effects) may have had some level of influence [31].

A debatable point is the decision to exclude those who responded with “do not know” to the question about readiness to be tested. However, in view of the overall sample size (*n* > 6000), this subgroup was small (*n* = 93). In additional sensitivity analyses (data not shown) this group was added to those who had rejected the test, based on the assumption that expression of indecision might have been due to social desirability rather than an actual conflicted motivation. The analysis did not indicate any change in results.

## 5. Conclusions

We were able to show that, underneath a generally high level of interest in personalized medicine, differences in the extent of potential acceptance exist. Beyond socio-demographic factors, it seems that it is mainly different types of personal vulnerability, but also more general behavioral dispositions, which drive this variation. It is important to note, however, that some of the differences were small, and differential acceptance rates occurred on the basis of an overall high level of positive interest. In all subgroups a majority remained in favor of accepting the testing offer. Additionally, due to the study’s spotlight on the older population segment, it has to remain open at this point to which extent results would also apply to the overall adult population.

Further, the current study focused exclusively on the role of individual factors, thus neglecting the role of situational differences. Perceived attractiveness of personalized medicine might thus also depend on characteristics of the diseases at stake (degree of severity and degree of treatability), on test and treatment characteristics (different types of benefits, such as increasing effectiveness versus avoiding mild or severe side-effects), on the specific risks involved with genetic testing (such as questions of data security and who has access to test results), and on the quality/accuracy of tests [31,72].

Future studies should therefore test the relative relevance of personal in relation to situational and test-related characteristics as well as investigate interactions between these factors. More information is also needed about the specific mediating mechanisms that make different subpopulations either more or less willing to accept testing. While it is, for instance, plausible that different worldviews about “omics-based technologies” and their financing within increasingly resource-challenged health care budgets play a role in the relation between socio-demographic characteristics and acceptance of personalized medicine their actual relevance requires testing by more complex study designs.

The fact that almost 40% of Danish 50–80-year-olds were not yet aware of personalized medicine indicates a need for broader and more systematic information to better prepare the public for decisions they might have to make at some point in the future. This finding clearly fits in with the results of a recent review, where Calabró et al. (2020) identified a definite need for more systematic education in this area. To avoid confusion among the public they particularly recommended more consistency in the use of the term “personalized medicine” [38]. Such a need for clarification was also stated by Botham et al. (2021) as a conclusion from their review on understanding of the term “personalized medicine” in cancer treatment [68]. In view of the present study’s finding about higher awareness being related to lower levels of testing readiness, it additionally seems necessary to explore country- and subpopulation-specific mental models and (mis-) conceptions among those who have heard about or have some prior (limited) knowledge about genetic testing and personalized medicine. Such concepts have been shown to influence receipt of further genetic information [69] but may also affect general willingness to be tested. Finding out about these mental models and how they shape the level of interest in personalized medicine will identify starting points for more systematic information and explanation by clinicians and public health authorities.

Specifically, it appears necessary to clarify the options and limitations of personalized medicine to the public and openly communicate about costs and benefits of testing in the context of different diseases/treatments. Specifically, societal consequences in terms of protection against misuse of data and/or distribution of costs, might need an intensified debate and clearer communication.

## Figures and Tables

**Table 1:** Sample characteristics: Study sample and gross sample of 50–80 years old citizens compared to the same-aged Danish population.

		Study Sample (*n* = 6695) (%)	Gross Sample ^1^ (*n* = 6807) (%)	DK Population (50–80 Years) (*n* = 2,054,477) (%)
**Sex**	Female	52.1	52.5	50.9
**Age**	50–60	38.8	38.8	42.3
	61–70	36.6	36.6	32.1
	71–80	24.5	24.6	25.6
**Education**	Less than 11 years school education	18.8	18.9	27.3
	11–13 years school education	44.1	44.0	43.8
	>13 years school education	37.1	37.1	28.9
**Personal income**	<27.000 €		34.9	42.3
	27.000–40.000 €		32.7	30.6
	>40.000 €		32.4	27.1
**Work status**	Working		51.7	48.3
**Birthplace**	Denmark		94.9	92.0
**Residence in DK**	Capital (Copenhagen)		28.7	28.3
	Zealand		16.1	16.3
	Jutland & Funen		55.2	55.3

^1^ All respondents who returned a completed questionnaire.

**Table 2:** Bivariate associations between sample characteristics and readiness to accept a genetic test for personalized treatment.

Characteristics	Willing to Be Tested		Not Willing to Be Tested		Total		
	N	%	N	%	N	%	*p*-Value
**Overall (row/%)**	**5331**	**79.6**	**1364**	**20.4**	**6695**	**100.00**	
**Sex**							
Male	2675	83.4	531	16.6	3206	47.9	
Female	2656	76.1	833	23.9	3489	52.1	<0.001
**Age**							
50–60	2139	82.3	461	17.7	2600	38.8	
61–70	1946	79.4	506	20.6	2452	36.6	
71–80	1246	75.8	397	24.2	1643	24.5	<0.001
**Education**							
<11 years	1011	80.1	251	19.9	1262	18.8	
11–13 years	2406	81.5	546	18.5	2952	44.1	
>13 years	1914	77.1	567	22.9	2481	37.1	<0.001
**Income in € p.a.** ^1^							
<33.333	1993	77.5	577	22.5	2570	38.4	
33.333–46.666	1606	80.7	384	19.3	1990	29.7	
>46.666	1732	81.1	403	18.9	2135	31.9	=0.004
**Smoking status**							
Smoker	770	78.6	210	21.4	980	14.6	
Quit smoking	1467	81.3	337	18.7	1804	26.9	
Never smoker	3094	79.1	817	20.9	3911	58.4	=0.105
**Alcohol consumption**							
0–7 (women)/0–14 (men) units	4297	79.5	1108	20.5	5405	80.7	
>7 (women)/>14 (men) units	1034	80.2	256	19.8	1290	19.3	=0.315
**Physical activity**							
Never to 4 times a week	4580	80.0	1147	20.0	5727	85.6	
5 times a week or more	749	77.7	215	22.3	964	14.4	=0.058
**Body mass index**							
<25.0	2120	77.3	623	22.7	2743	41.8	
25.0–<30.0	2053	80.5	498	19.5	2551	38.8	
30.0+	1069	83.8	207	16.2	1276	19.4	<0.001
**Daily medication intake**							
No	2136	79.0	568	21.0	2704	40.4	
Yes	3192	80.1	792	19.9	3984	59.6	=0.137
**Satisfaction with health**							
Low	536	84.4	99	15.6	635	9.5	
Medium	1063	81.5	242	18.5	1305	19.5	
High	3730	78.5	1023	21.5	4753	71.0	<0.001
**Genetic vulnerability**							
No	2312	76.2	723	23.8	3035	45.3	
Don’t know	2065	81.0	483	19.0	2548	38.1	<0.001
Yes	954	85.8	158	14.2	1112	16.6	
**Internal health locus of control**							
Low	2837	78.2	790	21.8	3627	54.6	
High	2457	81.3	564	18.7	3021	45.4	=0.002
**Health-related risk taking**							
None	1191	78.0	335	22.0	1526	22.8	
Low	2775	79.6	710	20.4	3485	52.1	
Medium-High	1360	81.2	315	18.8	1675	25.1	=0.087
**Prior awareness about personalized medicine**							
No	2137	85.2	371	14.8	2508	37.5	
Yes	3183	76.3	990	23.7	4173	62.5	<0.001

^1^ Equivalized disposable income p.a. (OECD Equivalence Scale: Personal income after taxes weighted by number of household members), rounded numbers in €.

**Table 3:** Logistic regression model for factors associated with readiness to accept a genetic test for personalized treatment (*n* = 6470 for the full model).

Characteristics	Crude OR	95% CI	Adjusted OR	95% CI
**Sex**				
Female	**0.63**	**0.56–0.72**	**0.67**	**0.59–0.77**
**Age group**				
50–60	1	Ref	1	Ref
61–70	**0.83**	**0.72–0.95**	0.88	0.76–1.02
71–80	**0.68**	**0.58–0.79**	**0.72**	**0.61–0.86**
**Education**				
<11 years	1	Ref	1	Ref
11–13 years	1.09	0.93–1.29	1.04	0.87–1.24
>13 years	**0.84**	**0.71–0.99**	0.84	0.70–1.02
**Income per year (€)** ^1^				
<33,000	1	Ref	1	Ref
33.000–47.000	**1.21**	**1.05–1.40**	**1.23**	**1.05–1.44**
>47.000	**1.24**	**1.08–1.44**	**1.29**	**1.09–1.52**
**Smoking status**				
Smoker	1	Ref	1	Ref
Quit smoking	1.19	0.98–1.44	**1.25**	**1.02–1.54**
Never smoked	1.03	0.87–1.23	1.19	0.99–1.43
**Alcohol consumption over recommended levels**				
>7 (women)/>14 units (men)	1.04	0.90–1.21	1.09	0.93–1.28
**Sufficient physical activity**				
5 days a week or more	0.87	0.74–1.03	0.95	0.80–1.13
**BMI**				
<25.0	1	Ref	1	Ref
25.0–29.9	**1.21**	**1.06–1.38**	1.05	0.92–1.21
=>30.0	1.52	1.28–1.81	1.24	1.03–1.49
**Daily medication intake**	1.07	0.95–1.21	1.03	0.89–1.18
**Satisfaction with health status**	1	Ref	1	Ref
Low	0.81	0.63–1.05	0.73	0.56–0.96
Medium	0.81	0.63–1.05	0.73	**0.56–0.96**
High	**0.67**	**0.54–0.84**	**0.64**	**0.49–0.82**
**Perceived genetic vulnerability**				
No	1	Ref	1	Ref
Don’t know	**1.34**	**1.17–1.52**	**1.38**	**1.20–1.58**
Yes	**1.89**	**1.57–2.28**	**1.91**	**1.57–2.34**
**Internal health locus of control**	**1.21**	**1.08–1.37**	**1.21**	**1.06–1.38**
**Health-related risk taking**				
None	1	Ref	1	Ref
Low	1.10	0.95–1.27	1.00	0.85–1.17
Medium-High	**1.21**	**1.02–1.44**	1.02	0.85–1.23
**Prior awareness about**				
**personalized medicine**	**0.56**	**0.49–0.64**	**0.58**	**0.50–0.66**
**Extraversion (1–7)**	**1.05**	**1.01–1.09**	**1.08**	**1.03–1.14**
**Openness (1–7)**	**0.99**	**0.94–1.04**	0.98	0.93–1.04
**Emotional Stability (1–7)**	**1.06**	**1.01–1.11**	**1.06**	**1.01–1.12**

^1^ Equivalized disposable income p.a. (OECD Equivalence Scale: Personal income after taxes weighted by number of household members), rounded numbers in €.

## Data Availability

The data presented in this study are available in aggregated form on request from the corresponding author. Data on individuals are not available due to EU GDPR regulation.
